# Supplementary material for: Use and experiences of galactagogues while breastfeeding among Australian women
Source: PLoS One. 2021 Jul 1;16(7):e0254049. doi: 10.1371/journal.pone.0254049 (PMC8248610; doi:10.1371/journal.pone.0254049)
Supplement: S1 File — All survey questions asked regarding women’s use and experiences with substances to boost breast milk supply. (PDF) [file pone.0254049.s001.pdf]

# Boosting Breast Milk Supply

**Participant Information Sheet**

**HUMAN RESEARCH ETHICS COMMITTEE APPROVAL NUMBER: H-2019-33934**
**PRINCIPAL INVESTIGATOR: Dr Luke Grzeskowiak**

**What is the project about?**
**We are conducting a survey to better understand what women know about taking medicines or other substances (such as herbs or foods) to boost breast milk supply.**

**Who is undertaking the project?**
**This project is being conducted by Dr Luke Grzeskowiak, Dr Gabbie Zizzo, Ms Robyn Stevenson and Associate Professor Alice Rumbold from the University of Adelaide. This project has been funded by the Robinson Research Institute at the University of Adelaide.**

**What am I being asked to do and how long will it take?**
**We ask that you complete the following survey that consists of a series of questions asking about yourself, demographics, the birth of your youngest child, your breastfeeding experience, and your awareness and use (if relevant) of medications or other substances to boost breast milk supply.**

**How much time will my involvement in the project take?**
**We estimate the survey will take 10-20 minutes to complete.**

**Are there any risks associated in participating in this study?**
**We recognise that asking questions about your birth or breastfeeding experience could be distressing to some women. The following support services are available if you need:**

**Breastfeeding support**
**Australian Breastfeeding Association - Breastfeeding Hotline - 1800 686 268**

**Bereavement Support**
**SANDS - Miscarriage, stillbirth, and newborn death support - 1300 072 637**

**Mental Health Support**
**Beyond Blue - 1300 224 636**
**Lifeline - 13 11 14**

**What are the potential benefits of the research project?**
**There are no immediate benefits to you for participating in this study. The information you provide could help us understand how to better support women when they are making decisions about whether to take a medication or other substance to boost their breast milk supply.**

**Can I withdraw from the project?**
**Completing the survey is voluntary. If you complete the survey anonymously, it will not be possible to withdraw your data once responses have been submitted. If you choose to provide your contact details when responding to the survey, you can ask to withdraw any or all of your data up to 1 month following submission of the survey by contacting the principal investigator: Dr Luke Grzeskowiak Email: luke.grzeskowiak@adelaide.edu.au OR Phone: (08) 8313 1687**

**What will happen to the data I provide?**
**Confidentiality and privacy: All responses will remain anonymous, unless you wish to provide your contact details to participate in a follow-up interview study to discuss your responses in more detail. Your responses will be de-identified prior to analysis. While all efforts will be made to remove any information that might identify you, as the sample size is small, complete anonymity cannot be guaranteed. However, the utmost care will be taken to ensure that no personally identifying details are revealed.**
**Storage: All Electronic records will be kept in password protected files on the University servers. Only those directly involved in the study will have access to the data. Data will be kept for 5 years.**
**Publishing: Information from this project will be used to generate a report, which will be presented at a conference and submitted for publication in a peer-reviewed journal. The report will also be submitted to the funding body. Participants will not be identified in any reports.**
**Sharing: Individual de-identified responses to the survey will be made available to other researchers on request for research projects that are in the same general area of research. Any identifying information will be kept seperate to your survey responses. Information collected during the survey may also be used by the same reserarchers in future research projects that are an extension of, or closely related to, the original project.**

**Who do I contact if I have questions about the project?**
**If you have any questions about the project you can contact any of the following members of the research team:**

**Dr Luke Grzeskowiak - Email: luke.grzeskowiak@adelaide.edu.au OR Phone: (08) 8313 1687**
**Associate Professor Alice Rumbold - Email: alice.rumbold@sahmri.com OR Phone: (08) 8128 4194**
**Dr Gabbie Zizzo - Email: gabriella.zizzo@adelaide.edu.au OR Phone: (08) 8313 0152**
**Ms Robyn Stevenson - Email: robyn.stevenson@student.adelaide.edu.au OR Phone: (08) 8313 1687**

**What if I have a complaint or any concerns?**
**The study has been approved by the Human Research Ethics Committee at the University of Adelaide (approval number H-2019-33934). This research project will be conducted according to the NHMRC National Statement on Ethical Conduct in Human Research 2007 (Updated 2018). If you have questions or problems associated with the practical aspects of your participation in the project, or wish to raise a concern or complaint about the project, then you should consult the Principal Investigator. If you wish to speak with an independent person regarding concerns or a complaint, the University's policy on research involving human**

**participants, or your rights as a participant, please contact the Human Research Ethics Committee's Secretariat on:**

**Phone: +61 8 8313 6028**
**Email: hrec@adelaide.edu.au**
**Post: Level 4, Rundle Mall Plaza, 50 Rundle Mall, ADELAIDE SA 5000**

**Any complaint or concern will be treated in confidence and fully investigated. You will be informed of the outcome.**

**Yours sincerely,**
**Dr Luke Grzeskowiak**
**Ms Robyn Stevenson**
**Dr Gabbie Zizzo**
**Associate Professor Alice Rumbold**

**Ver. 3.0**
**Date: 12/11/19**

If I want to participate, what do I do?
If you would like to participate in the study, continue to the next page. Submission of completed responses to the survey will be taken as an indication of consent to participate.

**Section 1**

| | |
|---|---|
| What is your current age? | ______________________ |
| Do you currently live in Australia? | ◯ Yes<br>◯ No |
| Were you born in Australia? | ◯ Yes<br>◯ No |
| Where were you born? | ______________________ |
| In which country was your father born? | ◯ Australia<br>◯ Other |
| If you selected other, please specify which country. | ______________________ |
| In which country was your mother born? | ◯ Australia<br>◯ Other |
| If you selected other, please specify which country. | ______________________ |
| Do you mainly speak English at home? | ◯ Yes<br>◯ No |

| | |
|---|---|
| What language do you mainly speak at home? | __________________________________ |
| Are you of Aboriginal or Torres Strait Islander origin? (For persons of both Aboriginal and Torres Strait Islander origin, mark both 'Yes' boxes) | ☐ No<br>☐ Yes, Aboriginal<br>☐ Yes, Torres Strait Islander<br>☐ Not sure/ prefer not to say |
| Did you complete secondary school? (year 12 or equivalent)? | ◯ Yes<br>◯ No<br>◯ Not sure |
| What is the highest level of education you have completed? | ◯ Yes - Certificate/Apprenticeship<br>◯ Yes - Diploma<br>◯ Yes - Degree (undergraduate or honours)<br>◯ Yes - Higher Degree (masters or doctorate)<br>◯ Yes - Other<br>◯ No<br>◯ Not Sure |
| If you selected other, please specify. | __________________________________ |
| Please tell us your current height | __________________________________<br>(cm) |
| Please tell us your current weight (optional) | __________________________________<br>(kg) |
| Which of the following have you heard can be used to increase breast milk supply? (tick all that apply) | ☐ domperidone (eg. Motilium ®)<br>☐ metoclopramide (eg. Maxolon®, Pramin®)<br>☐ fenugreek<br>☐ blessed thistle<br>☐ fennel<br>☐ milk thistle<br>☐ ginger<br>☐ brewer's yeast<br>☐ lactation cookies<br>☐ other<br>☐ none |
| If you selected other, please specify. | __________________________________ |

**On a scale of 1 to 5, how safe do you think each of the following are for MOTHERS when used to increase breast milk supply?**
**1 = Very Safe**
**2 = Somewhat Safe**
**3 = Not Sure**
**4 = Somewhat Unsafe**
**5 = Very Unsafe**

| | 1 | 2 | 3 | 4 | 5 |
|---|---|---|---|---|---|
| domperidone | ○ | ○ | ○ | ○ | ○ |
| metoclopramide | ○ | ○ | ○ | ○ | ○ |
| fenugreek | ○ | ○ | ○ | ○ | ○ |
| blessed thistle | ○ | ○ | ○ | ○ | ○ |
| fennel | ○ | ○ | ○ | ○ | ○ |
| milk thistle | ○ | ○ | ○ | ○ | ○ |
| ginger | ○ | ○ | ○ | ○ | ○ |
| brewer's yeast | ○ | ○ | ○ | ○ | ○ |
| lactation cookies | ○ | ○ | ○ | ○ | ○ |
| other substance you used | ○ | ○ | ○ | ○ | ○ |

**On a scale of 1 to 5, how safe do you think each of the following are for INFANTS when used to increase breast milk supply?**
**1 = Very Safe**
**2 = Somewhat Safe**
**3 = Not Sure**
**4 = Somewhat Unsafe**
**5 = Very Unsafe**

| | 1 | 2 | 3 | 4 | 5 |
|---|---|---|---|---|---|
| domperidone | ○ | ○ | ○ | ○ | ○ |
| metoclopramide | ○ | ○ | ○ | ○ | ○ |
| fenugreek | ○ | ○ | ○ | ○ | ○ |
| blessed thistle | ○ | ○ | ○ | ○ | ○ |
| fennel | ○ | ○ | ○ | ○ | ○ |
| milk thistle | ○ | ○ | ○ | ○ | ○ |
| ginger | ○ | ○ | ○ | ○ | ○ |
| brewer's yeast | ○ | ○ | ○ | ○ | ○ |
| lactation cookies | ○ | ○ | ○ | ○ | ○ |
| other substance you used | ○ | ○ | ○ | ○ | ○ |

| | |
|---|---|
| Which of the following sources have you used to obtain information on the use of medicines or other substances to increase breast milk supply (tick all that apply)? | ☐ None<br>☐ General Practitioner (GP)<br>☐ Obstetrician/Gynaecologist<br>☐ Neonatal doctor/Paediatrician<br>☐ Midwife<br>☐ Lactation Consultant<br>☐ Neonatal Nurse<br>☐ Community Pharmacist<br>☐ Child and Family Health Nurse<br>☐ Naturopath<br>☐ Breastfeeding Helpline<br>☐ Medicines Information Phone Line<br>☐ Family<br>☐ Friends<br>☐ Mother's Group (i.e. face-to-face)<br>☐ Internet Search (e.g. Google)<br>☐ Social Media (e.g. Instagram, Twitter, Facebook)<br>☐ Blogs or Online Discussion Forums<br>☐ Podcasts<br>☐ Books<br>☐ Other |
| If you selected Social Media, please specify your source. | ☐ Reddit<br>☐ Instagram<br>☐ Facebook<br>☐ Twitter<br>☐ Snapchat<br>☐ Other |
| If you selected other, please specify which information source. | __________________________________ |

**On a scale of 1 to 5, how useful were each of the following in helping you decide which medicine or substance to take to increase your breast milk supply?**
**1 = Very Helpful**
**2 = Somewhat Helpful**
**3 = Not Sure**
**4 = Somewhat Unhelpful**
**5 = Very Unhelpful**

| | 1 | 2 | 3 | 4 | 5 |
|---|---|---|---|---|---|
| General Practitioner (GP) | ◯ | ◯ | ◯ | ◯ | ◯ |
| Obstetrician/Gynaecologist | ◯ | ◯ | ◯ | ◯ | ◯ |
| Neonatal Doctor | ◯ | ◯ | ◯ | ◯ | ◯ |
| Midwife | ◯ | ◯ | ◯ | ◯ | ◯ |
| Lactation Consultant | ◯ | ◯ | ◯ | ◯ | ◯ |
| Neonatal Nurse | ◯ | ◯ | ◯ | ◯ | ◯ |
| Community Pharmacist | ◯ | ◯ | ◯ | ◯ | ◯ |
| Child and Family Health Nurse | ◯ | ◯ | ◯ | ◯ | ◯ |
| Naturopath | ◯ | ◯ | ◯ | ◯ | ◯ |
| Breastfeeding Helpline | ◯ | ◯ | ◯ | ◯ | ◯ |
| Medicines Information Phone Line | ◯ | ◯ | ◯ | ◯ | ◯ |
| Family | ◯ | ◯ | ◯ | ◯ | ◯ |
| Friends | ◯ | ◯ | ◯ | ◯ | ◯ |
| Mother's Group (e.g. face-to-face) | ◯ | ◯ | ◯ | ◯ | ◯ |
| Internet Search (e.g. Google) | ◯ | ◯ | ◯ | ◯ | ◯ |
| Social Media (e.g. Instagram, Twitter, Facebook) | ◯ | ◯ | ◯ | ◯ | ◯ |
| Blogs or Online Discussion Forums | ◯ | ◯ | ◯ | ◯ | ◯ |
| Podcasts | ◯ | ◯ | ◯ | ◯ | ◯ |
| Books | ◯ | ◯ | ◯ | ◯ | ◯ |
| Other Source You Mentioned Previously | ◯ | ◯ | ◯ | ◯ | ◯ |

| | |
|---|---|
| How important do you think it is for researchers to do research on helping mothers to increase their breast milk supply using medications or other substances? | ◯ Very Important<br>◯ Important<br>◯ Fairly Important<br>◯ Slightly Important<br>◯ Not Important |

| | |
|---|---|
| How many children do you have? | __________________________________ |
| The following questions relate to your youngest child. | |
| Did you give birth to multiples (e.g. twin, triplets, quadruplets)? | ◯ Yes ◯ No |
| When was your youngest child born? | __________________________________ |
| Where was your youngest child born? | ◯ SA ◯ QLD ◯ VIC<br>◯ NT ◯ TAS ◯ WA<br>◯ NSW ◯ ACT ◯ Outside of Australia |
| Did you give birth preterm (before 37 weeks' gestation)? | ◯ Yes ◯ No |
| How many weeks pregnant were you when you gave birth? | __________________________________<br>(weeks) |
| Was your child born by caesarean? | ◯ Yes<br>◯ No |
| Did you attend any classes or information sessions whilst pregnant that included information about breastfeeding? | ◯ Yes<br>◯ No, no need as this was not my first baby<br>◯ No, was not aware of any classes<br>◯ No, too expensive<br>◯ No, too hard to get to classes<br>◯ No, not interested or not the way I learn or get information<br>◯ No, other reasons |
| If you selected other reasons, please specify. | __________________________________ |
| Before giving birth, how did you plan to feed your child? | ◯ Breast milk only<br>◯ Mainly breast milk with some formula<br>◯ A somewhat equal mix of breast milk and formula<br>◯ Mainly formula with some breast milk<br>◯ Formula only<br>◯ Undecided |
| Can you remember how long you originally planned to breastfeed? | ◯ A set amount of months<br>◯ As long as I could<br>◯ Not sure / can't remember<br>◯ Didn't have a plan / set idea |
| How many months? | __________________________________<br>(Months) |

| | |
|---|---|
| Did you smoke while breastfeeding?<br>(includes cigarettes/ cigars/ vaping/ e-cigarettes)? | ◯ Yes, daily<br>◯ Yes, only occasionally (less than daily)<br>◯ No<br>◯ Not sure |
| Did you use nicotine replacement therapy (NRT) while breastfeeding? | ◯ Yes<br>◯ No<br>◯ Unsure |
| Did you ever feel that you could not make enough breast milk to feed your child? | ◯ Yes<br>◯ No |
| Did you get help from a lactation consultant or breastfeeding expert to help with breastfeeding? | ◯ Yes<br>◯ No |
| Did your child require supplementation with donor human milk? | ◯ Yes<br>◯ No |
| Did your child require supplementation with infant formula? | ◯ Yes<br>◯ No |
| Are you currently breastfeeding your child? | ◯ Yes<br>◯ No |
| How old is your child? | ______________________________<br>(months) |
| When did you stop breastfeeding? | ______________________________<br>(months) |
| Did you stop breastfeeding because of low milk supply? | ◯ Yes<br>◯ No |
| Did you use any medicines or other substances to increase your breast milk supply? | ◯ Yes<br>◯ No |
| Have you breastfed or provided breast milk before to other children? | ◯ Yes<br>◯ No, this is my first child<br>◯ No, I haven't breastfed my other children |

| Question | Response |
| --- | --- |
| Following the birth of your youngest child, did you ever take domperidone (also known as Motilium®) to boost breast milk supply? | ◯ Yes<br>◯ No |
| Who prescribed domperidone for you? | ☐ Neonatologist<br>☐ General Practitioner (GP)<br>☐ Obstetrician/Gynaecologist<br>☐ Midwife<br>☐ Other |
| If other, please specify. | __________________________________ |
| When did you start taking domperidone following birth? | ◯ < 7 days<br>◯ 7-13 days<br>◯ 2-4 weeks<br>◯ >4 weeks<br>◯ >1 month-3 months<br>◯ >3 months |
| What was the highest amount of domperidone that you took in one day? | ◯ 1 tablet 3 times a day<br>◯ 2 tablets 3 times a day<br>◯ Other |
| If other, please specify. | __________________________________ |
| Are you still using domperidone? | ◯ Yes<br>◯ No |
| How long have you been taking domperidone? | __________________________________<br>(Weeks) |
| How long did you take domperidone for? | __________________________________<br>(Weeks) |
| Did you stop taking domperidone because of side effects? | ◯ Yes<br>◯ No |
| How effective do you feel domperidone was in increasing your breast milk supply? | ◯ Extremely effective<br>◯ Very effective<br>◯ Moderately effective<br>◯ Slightly effective<br>◯ Not at all effective |
| Would you use domperidone again? | ◯ Yes<br>◯ No |

| | |
|---|---|
| Did you experience any of the following side effects while taking domperidone? | ☐ none<br>☐ headache<br>☐ nausea<br>☐ dry mouth<br>☐ stomach cramps<br>☐ heart palpitations / racing heart<br>☐ dizziness / fainting<br>☐ depression<br>☐ irritability<br>☐ weight gain<br>☐ fatigue<br>☐ involuntary movements / jerking<br>☐ skin rash<br>☐ other |
| If other, please specify. | __________________________________ |
| Would you recommend domperidone to a friend? | ○ Yes<br>○ No |
| Why would you not recommend domperidone? | ☐ Too expensive<br>☐ Side effects<br>☐ Does not work<br>☐ Other |
| If other, please specify. | __________________________________ |

| | |
|---|---|
| Following the birth of your youngest child, did you ever take metoclopramide (also known as Pramin® or Maxolon®) to boost breast milk supply? | ◯ Yes<br>◯ No |
| Who prescribed metoclopramide for you? | ☐ Neonatologist<br>☐ General Practitioner (GP)<br>☐ Obstetrician/Gynaecologist<br>☐ Midwife<br>☐ Other |
| If other, please specify. | __________________________________ |
| When did you start taking metoclopramide following birth? | ☐ < 7 days<br>☐ 7-13 days<br>☐ 2-4 weeks<br>☐ >4 weeks<br>☐ >1 month-3 months<br>☐ >3 months |
| What was the highest amount of metoclopramide that you took in one day? | ◯ 1 tablet, 3 times a day<br>◯ 2 tablets, 3 times a day<br>◯ Other |
| If other, please specify. | __________________________________ |
| Are you still using metoclopramide? | ◯ Yes<br>◯ No |
| How long have you been using metoclopramide for? | __________________________________<br>(Weeks) |
| How long did you use metoclopramide for? | __________________________________<br>(Weeks) |
| Did you experience any of the following side effects while taking metoclopramide? | ☐ none<br>☐ headache<br>☐ nausea<br>☐ dry mouth<br>☐ stomach cramps<br>☐ heart palpitations / racing heart<br>☐ dizziness / fainting<br>☐ depression<br>☐ irritability<br>☐ weight gain<br>☐ fatigue<br>☐ involuntary movements / jerking<br>☐ skin rash<br>☐ other |
| If other, please specify. | __________________________________ |

| | |
|---|---|
| Did you stop taking metoclopramide because of side effects? | ◯ Yes<br>◯ No |
| How effective do you feel metoclopramide was in increasing your breast milk supply? | ◯ Extremely effective<br>◯ Very effective<br>◯ Moderately effective<br>◯ Slightly effective<br>◯ Not at all effective |
| Would you use metoclopramide again? | ◯ Yes<br>◯ No |
| Would you recommend metoclopramide to a friend? | ◯ Yes<br>◯ No |
| Why would you not recommend taking metoclopramide? | ☐ Too expensive<br>☐ Side effects<br>☐ Does not work<br>☐ Other |
| If other, please specify. | __________________________ |

| | |
|---|---|
| Following the birth of your youngest child, did you ever take any of the following herbal medications or a natural substances to boost your milk supply? | ☐ None<br>☐ Fenugreek<br>☐ Blessed thistle<br>☐ Fennel<br>☐ Milk thistle<br>☐ Ginger<br>☐ Brewer's yeast<br>☐ Lactation cookies<br>☐ Combination of multiple herbs<br>☐ Other |
| If you took a combination of herbs or natural substances, please specify. | __________________________________ |
| If other, please specify. | __________________________________ |

| | |
|---|---|
| Who recommended that you use fenugreek? | ☐ General Practitioner (GP)<br>☐ Midwife<br>☐ Lactation Consultant<br>☐ Neonatal Nurse<br>☐ Obstetrician/Gynaecologist<br>☐ Neonatal doctor/Paediatrician<br>☐ Community Pharmacist<br>☐ Naturopath<br>☐ Family<br>☐ Friends<br>☐ Child and Family Health Nurse<br>☐ Breastfeeding Helpline<br>☐ Mother's Group (i.e. face-to-face)<br>☐ Internet<br>☐ Social Media (e.g. Instagram, Twitter, Facebook)<br>☐ Blogs or Online Discussion Forums<br>☐ Podcast<br>☐ Book<br>☐ Other |
| If other, please specify. | __________________________ |
| When did you start taking fenugreek following birth? | ◯ < 7 days following birth<br>◯ 7-13 days<br>◯ 2-4 weeks<br>◯ >4 weeks<br>◯ >1 month-3 months<br>◯ >3 months |
| Are you still using fenugreek? | ◯ Yes<br>◯ No |
| How long have you been using fenugreek for? | __________________________<br>(Weeks) |
| How long did you take fenugreek for? | __________________________<br>(Weeks) |
| Did you experience any of the following side effects while taking fenugreek? | ☐ none<br>☐ headache<br>☐ nausea<br>☐ dry mouth<br>☐ stomach cramps<br>☐ heart palpitations / racing heart<br>☐ dizziness / fainting<br>☐ depression<br>☐ irritability<br>☐ weight gain<br>☐ fatigue<br>☐ involuntary movements / jerking<br>☐ skin rash<br>☐ other |
| If other, please specify. | __________________________ |

| | |
|---|---|
| How effective do you feel that fenugreek was in increasing your breast milk supply? | ◯ Extremely effective<br>◯ Very effective<br>◯ Moderately effective<br>◯ Slightly effective<br>◯ Not at all effective |
| Would you use fenugreek again? | ◯ Yes<br>◯ No |
| Would you recommend fenugreek to a friend? | ◯ Yes<br>◯ No |
| Why would you not recommend fenugreek? | ☐ Too expensive<br>☐ Side effects<br>☐ Does not work<br>☐ Other |
| If other, please specify. | __________________________ |

| | |
|---|---|
| Who recommended that you use blessed thistle? | ☐ General Practitioner (GP)<br>☐ Midwife<br>☐ Lactation Consultant<br>☐ Neonatal Nurse<br>☐ Obstetrician/Gynaecologist<br>☐ Neonatal doctor/Paediatrician<br>☐ Community Pharmacist<br>☐ Naturopath<br>☐ Family<br>☐ Friends<br>☐ Child and Family Health Nurse<br>☐ Breastfeeding Helpline<br>☐ Mother's Group (i.e. face-to-face)<br>☐ Internet<br>☐ Social Media (e.g. Instagram, Twitter, Facebook)<br>☐ Blogs or Online Discussion Forums<br>☐ Podcast<br>☐ Book<br>☐ Other |
| If other, please specify. | __________________________________ |
| When did you start taking blessed thistle following birth? | ◯ < 7 days<br>◯ 7-13 days<br>◯ 2-4 weeks<br>◯ >4 weeks<br>◯ >1 month-3 months<br>◯ >3 months |
| Are you still using blessed thistle | ◯ Yes<br>◯ No |
| How long have you been using blessed thistle for? | __________________________________<br>(Weeks) |
| How long did you take blessed thistle for? | __________________________________<br>(Weeks) |
| Did you experience any of the following side effects while taking blessed thistle? | ☐ none<br>☐ headache<br>☐ nausea<br>☐ dry mouth<br>☐ stomach cramps<br>☐ heart palpitations / racing heart<br>☐ dizziness / fainting<br>☐ depression<br>☐ irritability<br>☐ weight gain<br>☐ fatigue<br>☐ involuntary movements / jerking<br>☐ skin rash<br>☐ other |
| If other, please specify. | __________________________________ |

| | |
|---|---|
| How effective do you feel blessed thistle was in increasing your breast milk supply? | ◯ Extremely effective<br>◯ Very effective<br>◯ Moderately effective<br>◯ Slightly effective<br>◯ Not at all effective |
| Would you use blessed thistle again? | ◯ Yes<br>◯ No |
| Would you recommend blessed thistle to a friend? | ◯ Yes<br>◯ No |
| Why would you not recommend blessed thistle? | ☐ Too expensive<br>☐ Side effects<br>☐ Does not work<br>☐ Other |
| If other, please specify. | __________________________ |

| | |
|---|---|
| Who recommended that you use fennel? | ☐ General Practitioner (GP)<br>☐ Midwife<br>☐ Lactation Consultant<br>☐ Neonatal Nurse<br>☐ Obstetrician/Gynaecologist<br>☐ Neonatal doctor/Paediatrician<br>☐ Community Pharmacist<br>☐ Naturopath<br>☐ Family<br>☐ Friends<br>☐ Child and Family Health Nurse<br>☐ Breastfeeding Helpline<br>☐ Mother's Group (i.e. face-to-face)<br>☐ Internet<br>☐ Social Media (e.g. Instagram, Twitter, Facebook)<br>☐ Blogs or Online Discussion Forums<br>☐ Podcast<br>☐ Book<br>☐ Other |
| If other, please specify. | __________________________ |
| When did you start taking fennel following birth? | ◯ < 7 days<br>◯ 7-13 days<br>◯ 2-4 weeks<br>◯ >4 weeks<br>◯ >1 month-3 months<br>◯ >3 months |
| Are you still using fennel? | ◯ Yes<br>◯ No |
| How long have you been using fennel for? | __________________________<br>(Weeks) |
| How long did you take fennel for? | __________________________<br>(Weeks) |
| Did you experience any of the following side effects while taking fennel? | ☐ none<br>☐ headache<br>☐ nausea<br>☐ dry mouth<br>☐ stomach cramps<br>☐ heart palpitations / racing heart<br>☐ dizziness / fainting<br>☐ depression<br>☐ irritability<br>☐ weight gain<br>☐ fatigue<br>☐ involuntary movements / jerking<br>☐ skin rash<br>☐ other |
| If other, please specify. | __________________________ |

| | |
|---|---|
| How effective do you feel fennel was in increasing your breast milk supply? | ◯ Extremely effective<br>◯ Very effective<br>◯ Moderately effective<br>◯ Slightly effective<br>◯ Not at all effective |
| Would you use fennel again? | ◯ Yes<br>◯ No |
| Would you recommend fennel to a friend? | ◯ Yes<br>◯ No |
| Why would you not recommend fennel? | ☐ Too expensive<br>☐ Side effects<br>☐ Does not work<br>☐ Other |
| If other, please specify. | ________________________ |

| Question | Response |
| --- | --- |
| Who recommended that you use milk thistle? | ☐ General Practitioner (GP)<br>☐ Midwife<br>☐ Lactation Consultant<br>☐ Neonatal Nurse<br>☐ Obstetrician/Gynaecologist<br>☐ Neonatal doctor/Paediatrician<br>☐ Community Pharmacist<br>☐ Naturopath<br>☐ Family<br>☐ Friends<br>☐ Child and Family Health Nurse<br>☐ Breastfeeding Helpline<br>☐ Mother's Group (i.e. face-to-face)<br>☐ Internet<br>☐ Social Media (e.g. Instagram, Twitter, Facebook)<br>☐ Blogs or Online Discussion Forums<br>☐ Podcast<br>☐ Book<br>☐ Other |
| If other, please specify. | ________________________ |
| When did you start taking milk thistle following birth? | ○ < 7 days<br>○ 7-13 days<br>○ 2-4 weeks<br>○ >4 weeks<br>○ >1 month-3 months<br>○ >3 months |
| Are you still using milk thistle? | ○ Yes<br>○ No |
| How long have you been using milk thistle for? | ________________________<br>(Weeks) |
| How long did you take milk thistle for? | ________________________<br>(Weeks) |
| Did you experience any of the following side effects while taking milk thistle? | ☐ none<br>☐ headache<br>☐ nausea<br>☐ dry mouth<br>☐ stomach cramps<br>☐ heart palpitations / racing heart<br>☐ dizziness / fainting<br>☐ depression<br>☐ irritability<br>☐ weight gain<br>☐ fatigue<br>☐ involuntary movements / jerking<br>☐ skin rash<br>☐ other |
| If other, please specify. | ________________________ |

| | |
|---|---|
| How effective do you feel milk thistle was in increasing your breast milk supply? | ◯ Extremely effective<br>◯ Very effective<br>◯ Moderately effective<br>◯ Slightly effective<br>◯ Not at all effective |
| Would you use milk thistle again? | ◯ Yes<br>◯ No |
| Would you recommend milk thistle to a friend? | ◯ Yes<br>◯ No |
| Why would you not recommend milk thistle? | ☐ Too expensive<br>☐ Side effects<br>☐ Does not work<br>☐ Other |
| If other, please specify. | __________________________ |

| | |
|---|---|
| Who recommended that you use ginger? | ☐ General Practitioner (GP)<br>☐ Midwife<br>☐ Lactation Consultant<br>☐ Neonatal Nurse<br>☐ Obstetrician/Gynaecologist<br>☐ Neonatal doctor/Paediatrician<br>☐ Community Pharmacist<br>☐ Naturopath<br>☐ Family<br>☐ Friends<br>☐ Child and Family Health Nurse<br>☐ Breastfeeding Helpline<br>☐ Mother's Group (i.e. face-to-face)<br>☐ Internet<br>☐ Social Media (e.g. Instagram, Twitter, Facebook)<br>☐ Blogs or Online Discussion Forums<br>☐ Podcast<br>☐ Book<br>☐ Other |
| If other, please specify. | ______________________ |
| When did you start taking ginger following birth? | ◯ < 7 days<br>◯ 7-13 days<br>◯ 2-4 weeks<br>◯ >4 weeks<br>◯ >1 month-3 months<br>◯ >3 months |
| Are you still using ginger? | ◯ Yes<br>◯ No |
| How long have you been using ginger for? | ______________________<br>(Weeks) |
| How long did you take ginger for? | ______________________<br>(Weeks) |
| Did you experience any of the following side effects while taking ginger? | ☐ none<br>☐ headache<br>☐ nausea<br>☐ dry mouth<br>☐ stomach cramps<br>☐ heart palpitations / racing heart<br>☐ dizziness / fainting<br>☐ depression<br>☐ irritability<br>☐ weight gain<br>☐ fatigue<br>☐ involuntary movements / jerking<br>☐ skin rash<br>☐ other |
| If other, please specify. | ______________________ |

| | |
|---|---|
| How effective do you feel ginger was in increasing your breast milk supply? | ◯ Extremely effective<br>◯ Very effective<br>◯ Moderately effective<br>◯ Slightly effective<br>◯ Not at all effective |
| Would you use ginger again? | ◯ Yes<br>◯ No |
| Would you recommend ginger to a friend? | ◯ Yes<br>◯ No |
| Why would you not recommend ginger? | ☐ Too expensive<br>☐ Side effects<br>☐ Does not work<br>☐ Other |
| If other, please specify. | __________________________________ |

Who recommended that you use brewer's yeast?

- ☐ General Practitioner (GP)
- ☐ Midwife
- ☐ Lactation Consultant
- ☐ Neonatal Nurse
- ☐ Obstetrician/Gynaecologist
- ☐ Neonatal doctor/Paediatrician
- ☐ Community Pharmacist
- ☐ Naturopath
- ☐ Family
- ☐ Friends
- ☐ Child and Family Health Nurse
- ☐ Breastfeeding Helpline
- ☐ Mother's Group (i.e. face-to-face)
- ☐ Internet
- ☐ Social Media (e.g. Instagram, Twitter, Facebook)
- ☐ Blogs or Online Discussion Forums
- ☐ Podcast
- ☐ Book
- ☐ Other

If other, please specify.

______________________________

When did you start taking brewer's yeast following birth?

- ◯ < 7 days
- ◯ 7-13 days
- ◯ 2-4 weeks
- ◯ >4 weeks
- ◯ >1 month-3 months
- ◯ >3 months

Are you still using brewer's yeast?

- ◯ Yes
- ◯ No

How long have you been using brewer's yeast?

______________________________
(Weeks)

How long did you take brewer's yeast for?

______________________________
(Weeks)

Did you experience any of the following side effects while taking brewer's yeast?

- ☐ none
- ☐ headache
- ☐ nausea
- ☐ dry mouth
- ☐ stomach cramps
- ☐ heart palpitations / racing heart
- ☐ dizziness / fainting
- ☐ depression
- ☐ irritability
- ☐ weight gain
- ☐ fatigue
- ☐ involuntary movements / jerking
- ☐ skin rash
- ☐ other

If other, please specify.

______________________________

| | |
|---|---|
| How effective do you feel brewer's yeast was in increasing your breast milk supply? | ◯ Extremely effective<br>◯ Very effective<br>◯ Moderately effective<br>◯ Slightly effective<br>◯ Not at all effective |
| Would you use brewer's yeast again? | ◯ Yes<br>◯ No |
| Would you recommend brewer's yeast to a friend? | ◯ Yes<br>◯ No |
| Why would you not recommend brewer's yeast? | ☐ Too expensive<br>☐ Side effects<br>☐ Does not work<br>☐ Other |
| If Other, please specify | __________________________________ |

| | |
|---|---|
| Who recommended that you use lactation cookies? | ☐ General Practitioner (GP)<br>☐ Midwife<br>☐ Lactation Consultant<br>☐ Neonatal Nurse<br>☐ Obstetrician/Gynaecologist<br>☐ Neonatal doctor/Paediatrician<br>☐ Community Pharmacist<br>☐ Naturopath<br>☐ Family<br>☐ Friends<br>☐ Child and Family Health Nurse<br>☐ Breastfeeding Helpline<br>☐ Mother's Group (i.e. face-to-face)<br>☐ Internet<br>☐ Social Media (e.g. Instagram, Twitter, Facebook)<br>☐ Blogs or Online Discussion Forums<br>☐ Podcast<br>☐ Book<br>☐ Other |
| If other, please specify. | __________________________ |
| When did you start taking lactation cookies following birth? | ◯ < 7 days<br>◯ 7-13 days<br>◯ 2-4 weeks<br>◯ >4 weeks<br>◯ >1 month-3 months<br>◯ >3 months |
| Are you still using lactation cookies? | ◯ Yes<br>◯ No |
| How long have you been using lactation cookies for? | __________________________<br>(Weeks) |
| How long did you take lactation cookies for? | __________________________<br>(Weeks) |
| Did you experience any of the following side effects while taking lactation cookies? | ☐ none<br>☐ headache<br>☐ nausea<br>☐ dry mouth<br>☐ stomach cramps<br>☐ heart palpitations / racing heart<br>☐ dizziness / fainting<br>☐ depression<br>☐ irritability<br>☐ weight gain<br>☐ fatigue<br>☐ involuntary movements / jerking<br>☐ skin rash<br>☐ other |
| If other, please specify. | __________________________ |

| | |
|---|---|
| How effective do you feel lactation cookies were in increasing your breast milk supply? | ◯ Extremely effective<br>◯ Very effective<br>◯ Moderately effective<br>◯ Slightly effective<br>◯ Not at all effective |
| Would you use lactation cookies again? | ◯ Yes<br>◯ No |
| Would you recommend lactation cookies to a friend? | ◯ Yes<br>◯ No |
| Why would you not recommend lactation cookies? | ☐ Too expensive<br>☐ Side effects<br>☐ Does not work<br>☐ Other |
| If other, please specify. | __________________________________ |

| | |
|---|---|
| Who recommended that you use this combination of herbs? | ☐ General Practitioner (GP)<br>☐ Midwife<br>☐ Lactation Consultant<br>☐ Neonatal Nurse<br>☐ Obstetrician/Gynaecologist<br>☐ Neonatal doctor/Paediatrician<br>☐ Community Pharmacist<br>☐ Naturopath<br>☐ Family<br>☐ Friends<br>☐ Child and Family Health Nurse<br>☐ Breastfeeding Helpline<br>☐ Mother's Group (i.e. face-to-face)<br>☐ Internet<br>☐ Social Media (e.g. Instagram, Twitter, Facebook)<br>☐ Blogs or Online Discussion Forums<br>☐ Podcast<br>☐ Book<br>☐ Other |
| If other, please specify. | __________________________________ |
| When did you start taking this combination of herbs following birth? | ◯ < 7 days<br>◯ 7-13 days<br>◯ 2-4 weeks<br>◯ >4 weeks<br>◯ >1 month-3 months<br>◯ >3 months |
| Are you still using this combination of herbs? | ◯ Yes<br>◯ No |
| How long have you been taking this combination of herbs for? | __________________________________<br>(Weeks) |
| How long did you take this combination of herbs for? | __________________________________<br>(Weeks) |
| Did you experience any of the following side effects while taking the combination of herbs? | ☐ none<br>☐ headache<br>☐ nausea<br>☐ dry mouth<br>☐ stomach cramps<br>☐ heart palpitations / racing heart<br>☐ dizziness / fainting<br>☐ depression<br>☐ irritability<br>☐ weight gain<br>☐ fatigue<br>☐ involuntary movements / jerking<br>☐ skin rash<br>☐ other |
| If other, please specify. | __________________________________ |

| | |
|---|---|
| How effective do you feel the combination of herbs was in increasing your breast milk supply? | ◯ Extremely effective<br>◯ Very effective<br>◯ Moderately effective<br>◯ Slightly effective<br>◯ Not at all effective |
| Would you use this combination of herbs again? | ◯ Yes<br>◯ No |
| Would you recommend this combination of herbs to a friend? | ◯ Yes<br>◯ No |
| Why would you not recommend this combination of herbs? | ☐ Too expensive<br>☐ Side effects<br>☐ Does not work<br>☐ Other |
| If other, please specify. | __________________________________ |

| | |
|---|---|
| Who recommended that you use the other substance you reported using? | ☐ General Practitioner (GP)<br>☐ Midwife<br>☐ Lactation Consultant<br>☐ Neonatal Nurse<br>☐ Obstetrician/Gynaecologist<br>☐ Neonatal doctor/Paediatrician<br>☐ Community Pharmacist<br>☐ Naturopath<br>☐ Family<br>☐ Friends<br>☐ Child and Family Health Nurse<br>☐ Breastfeeding Helpline<br>☐ Mother's Group (i.e. face-to-face)<br>☐ Internet<br>☐ Social Media (e.g. Instagram, Twitter, Facebook)<br>☐ Blogs or Online Discussion Forums<br>☐ Podcast<br>☐ Book<br>☐ Other |
| If other, please specify. | __________________________ |
| When did you start taking this following birth? | ◯ < 7 days<br>◯ 7-13 days<br>◯ 2-4 weeks<br>◯ >4 weeks<br>◯ >1 month-3 months<br>◯ >3 months |
| Are you still taking it? | ◯ Yes<br>◯ No |
| How long have you been using this for? | __________________________<br>(Weeks) |
| How long did you take this for? | __________________________<br>(Weeks) |
| Did you experience any of the following side effects while taking this? | ☐ none<br>☐ headache<br>☐ nausea<br>☐ dry mouth<br>☐ stomach cramps<br>☐ heart palpitations / racing heart<br>☐ dizziness / fainting<br>☐ depression<br>☐ irritability<br>☐ weight gain<br>☐ fatigue<br>☐ involuntary movements / jerking<br>☐ skin rash<br>☐ other |
| If other, please specify. | __________________________ |

| | |
|---|---|
| How effective do you feel this was in increasing your breast milk supply? | ◯ Extremely effective<br>◯ Very effective<br>◯ Moderately effective<br>◯ Slightly effective<br>◯ Not at all effective |
| Would you use this again? | ◯ Yes<br>◯ No |
| Would you recommend this to a friend? | ◯ Yes<br>◯ No |
| Why would you not recommend it? | ☐ Too expensive<br>☐ Side effects<br>☐ Does not work<br>☐ Other |
| If other, please specify. | __________________________________ |

This is the last question.

Do you have anything else that you would like to tell us about your thoughts or experiences of using medications or other substances for increasing breast milk supply? Feel free to write as much as you like.

__________________________________

**Thank you for completing this survey.**

**Your responses will help us to better inform women about different approaches towards improving their breast milk supply.**

**We welcome you to share this survey with any family, friends or colleagues who you think might be willing to share their experiences of considering medications and other substances for boosting breast milk supply. Given the nature of the questions asked, if you do share the survey, we ask that you do so in a sensitive manner that does not cause others embarrassment or distress.**

**If you would like to share this survey, please email this survey link: http://j.mp/2Q8bkNR**

**Additional research study**

**We would like to undertake interviews with women to further explore perceptions regarding the use of medicines and other substances for increasing breast milk supply. The interviews will either be completed in-person or over the phone, and are expected to take 30-45 minutes.**

**Detailed information regarding this study can be found below:**

Information sheet  for interviews

[Attachment: "Patient Information Sheet - Interviews.pdf"]

If you are interested in participating in a further study, please provide your contact details below:

Name: ________________________________

Email: ________________________________

Contact number: ________________________________
