# Supplementary material for: Use and experiences of galactagogues while breastfeeding among Australian women
Source: PLoS One. 2021 Jul 1;16(7):e0254049. doi: 10.1371/journal.pone.0254049 (PMC8248610; doi:10.1371/journal.pone.0254049)
Supplement: Attachment — Submitted filename: PLOS ONE Response to Reviewers_v1.docx [file pone.0254049.s002.docx]

Dear Editor,

Thank you for the feedback and for the opportunity to revise the manuscript.

We value the opportunity to further improve this manuscript with the following changes outlined below. Line numbers correspond to line numbers in the tracked changes copy of the document.

Additional Editor Comments:

1. **As requested by reviewer 1, please identify the relevant ingredients in ‘lactation cookies’.**

*Response 1: The following text has been added at line 68; ‘Anecdotally, recent studies demonstrate widespread awareness and use of galactagogues such as lactation cookies (Zizzo et al 2021). An examination of widely promoted recipes and commercially available products indicates that lactation cookies contain highly variable combinations and quantities of ingredients, including oats, brewer’s yeast and flaxseed.’, and line 359 regarding lactation cookies; ‘Lactation cookies were provided as one of ten listed galactagogues that women could choose from. However participants were not provided with a definition of lactation cookies nor a pre-defined list of ingredients. As such, our survey did not account for possible variations in ingredients between different lactation cookies, and there is likely to be some crossover with other galactagogues listed in the survey, particularly brewer’s yeast which is one of the most common ingredients in lactation cookies.’*

2. **Please carefully edit the methods section to avoid unnecessary repetition. For instance, reference to the use of R Upset Package is made on line 135 and then again on line 138. Can these sentences be combined to avoid repetition? Similarly, the method for reporting perceived effectiveness of galactagogues is first described on lines 105-107 and then again on lines 140-141.**

*Response 2: The repeating use of ‘R Upset package’ terms in line 157 has been amended to; ‘The most common combinations of galactagogues used were graphed using an UpSet plot.’*

*The repeating phrase on line 160-161 has been removed, and line 119-121 amended to; ‘ The perceived effectiveness of galactagogues was assessed using a 5-point Likert 5-point Likert scale from 1 (Not at all effective) to 5 (Extremely effective).’*

3. **Line 144 data is the plural of datum should read ‘data were’. Please check for any other instances.**

*Response 3: This error has been amended in text (line 164), and no other instances were found.*

4. **Table 1 p values of 0.000 should be reported as p<0.001**

*Response 4: This has been amended in the Table 1.*

5. **The sentence started on line 198 is incomplete**

*Response 5: The following amendment has been made to line 220, where the underlined text has been added; ‘The proportion of women reporting commencing individual galactagogues within the first seven days postpartum, ranged from 4 to 67%.’*

6. **There is some ambiguity related to the statement made in line 251.**

**Where do you provide the evidence for the statement that 1 in 5 women discontinued breastfeeding before they desired? In lines 159-160 you report “Of women who had stopped breastfeeding prior to completing the survey (35%), 19% reported stopping due to low milk supply.” In which case the 35% of women who stopped prior to completing the survey may have stopped for this reason but not all may necessarily have stopped prior to when they wanted to.**

**Furthermore, the statement "with almost 1 in 5 women discontinuing breastfeeding earlier than  desired because of concerns about their milk supply” is ambiguous and implies that 1 in 5 of the study sample discontinued breastfeeding earlier than desired for this reason  rather than 1 in 5 women who discontinued breastfeeding earlier than desired gave this reason for stopping.  Is this statement based on the results reported on lines 159-160?**

*Response 6: This statement has now been removed. Unfortunately we did not ask about whether or not breastfeeding was discontinued earlier than desired and are therefore not in a position to clarify this ambiguity.*

**Journal Requirements:
Please review your reference list to ensure that it is complete and correct. If you have cited papers that have been retracted, please include the rationale for doing so in the manuscript text, or remove these references and replace them with relevant current references. Any changes to the reference list should be mentioned in the rebuttal letter that accompanies your revised manuscript. If you need to cite a retracted article, indicate the article’s retracted status in the References list and also include a citation and full reference for the retraction notice.**

**When submitting your revision, we need you to address these additional requirements.**

*Two additional citations have been added during the revision process, the first is:*

*11.	Zizzo G, Amir LH, Moore V, Grzeskowiak LE, Rumbold AR. The risk-risk trade-offs: Understanding factors that influence women's decision to use substances to boost breast milk supply. PLoS One. 2021;16(5):e0249599.*

*This is a recent publication since the original paper was submitted, which demonstrates women’s use of lactation cookies, to support response one to the editor. The next paper added was:*

*9.	Morton J, Hall JY, Wong RJ, Thairu L, Benitz WE, Rhine WD. Combining hand techniques with electric pumping increases milk production in mothers of preterm infants. J Perinatol. 2009;29(11):757-64.*

*This reference was added to support response 2 to reviewer 2.*

**2. Please include additional information regarding the survey or questionnaire used in the study and ensure that you have provided sufficient details that others could replicate the analyses. For instance, if you developed a questionnaire as part of this study and it is not under a copyright more restrictive than CC-BY, please include a copy, in both the original language and English, as Supporting Information. If the original language is written in non-Latin characters, for example Amharic, Chinese, or Korean, please use a file format that ensures these characters are visible.**

*Response: The following has been added at line 134; ‘The complete survey is available as a supporting file (SI File 1).’ And a copy of the survey will be uploaded as a supporting file.*

**3. Please state whether you validated the questionnaire prior to testing on study participants. Please provide details regarding the validation group within the methods section.**

*Response: The following has been added at line 121-123; ‘The survey was tested for face validity with two consumers and an academic breastfeeding expert. Only minor changes were made to the survey before formal distribution…’*

**4. We note that you have indicated that data from this study are available upon request. PLOS only allows data to be available upon request if there are legal or ethical restrictions on sharing data publicly. For information on unacceptable data access restrictions, please see** [**http://journals.plos.org/plosone/s/data-availability#loc-unacceptable-data-access-restrictions**](http://journals.plos.org/plosone/s/data-availability#loc-unacceptable-data-access-restrictions)**.**

**a) If there are ethical or legal restrictions on sharing a de-identified data set, please explain them in detail (e.g., data contain potentially identifying or sensitive patient information) and who has imposed them (e.g., an ethics committee). Please also provide contact information for a data access committee, ethics committee, or other institutional body to which data requests may be sent.**

*Data cannot be shared publicly because the ethics committee restricts secondary use of the data currently. Data are available from The University of Adelaide Human Research Ethics Committee (contact T: +61 8 8313 5137; F: +61 8 8313 3700; research.services@adelaide.edu.au) for researchers who meet the criteria for access to confidential data.*

**Reviewers' comments:**

**Reviewer #1: A well written paper with important information about the use of galactagogues.
Just a few points for consideration:

1. Probably typo errors in the sentences in Lines 137-138 “The most common combinations of galactagogue use were graphed using the ‘UpSetR’ package in R.” and Lines 198-199 “The proportion of women reporting commencing individual galactagogues within the first seven days postpartum, ranged from.”**

*Response 1: The following amendment has been made to line 157, ‘The most common combinations of galactagogues used were graphed using an UpSet plot.’*

*The following amendment has been made to line 218-220, where the underlined text has been added; ‘The proportion of women reporting commencing individual galactagogues within the first seven days postpartum, ranged from 4 to 67%.’*
**2. Perhaps the authors could consider editing the sentence in Lines 71-73 to make it easier to understand? Also would the authors consider improving the way the two sentences in Lines 182-185 flowed?**

*Response 2: The following change has been made to lines 80-84; ‘Domperidone use at doses above 30 mg daily may present a risk of serious cardiac side effects (12). However the relevance to breastfeeding women has been questioned as previous data on increased cardiac risks mainly involved males and those aged over 60 years (13).’*

*The following change has been made to lines 202-205; ‘The most common patterns of galactagogue use are represented in Figure 1. Lactation cookies featured in the top five different combinations of galactagogues used, and were the most used sole galactagogue.’*

**3. With reference to Lines 130-132: Were there many participants who withdrew their responses when contacted during the separate qualitative study? Were there a lot of duplicate entries found during the search? A flow diagram capturing these details might be useful.**

*Response 3: The following amendment to line 146-151 have been made; ‘Only those who provided their contact details were able to withdraw their responses, however none elected to withdraw their responses. A total of 2152 responses were received, 7 responses were removed due to suspected duplicate entries based on identical maternal characteristics provided in the entry section, and a further 90 were removed due to births occurring outside of Australia.’*

**4. The data (percentages) in lines 211 to 215 is somewhat confusing to me. I could not tie this with the figures reported in Table 3.**

*Response 4: The line in Table 3 has been changed to reflect ‘Any side effects’ rather than ‘No side effects’ and following has been changed in line 234-235; ‘Domperidone had the highest proportion of women reporting one or more side effects (45%),’*

**5. What are lactation cookies made of? Perhaps some information about lactation cookies and the possible ingredients in the cookies that could have acted as a galactagogue could be mentioned in the background.**
*Response 5: As above in response 1:*

*The following text has been added at line 68; ‘Anecdotally, recent studies demonstrate widespread awareness and use of galactagogues such as lactation cookies (Zizzo et al 2021). An examination of widely promoted recipes and commercially available products indicates that lactation cookies contain highly variable combinations and quantities of ingredients, including oats, brewer’s yeast and flaxseed.’, and line 359 regarding lactation cookies; ‘Lactation cookies were provided as one of ten listed galactagogues that women could choose from. However participants were not provided with a definition of lactation cookies nor a pre-defined list of ingredients. As such, our survey did not account for possible variations in ingredients between different lactation cookies, and there is likely to be some crossover with other galactagogues listed in the survey, particularly brewer’s yeast which is one of the most common ingredients in lactation cookies.’*

**6. Lines 244 -248: Perhaps an emphasis on the need for properly conducted and properly described randomized controlled studies could be added after the call for future research? The main reason why there are extremely limited evidence on galactagogue efficacy is not because studies have not been done (there are over 100 studies as of year 2020), but there were problems with the research methods used (many were just observational studies) and the way the research was reported/described which prevented us from drawing firm findings. Another huge weakness of most studies is the lack of exploration of potential side effects, as well as long-term breastfeeding outcomes. (Lines 67-68 mentioned that “The review found uncertain evidence that galactagogues improve ……. longer-term breastfeeding outcomes” The lack of evidence here indeed was because there were no studies exploring this outcome.)**

*Response 6: An amendment has been made to line 388-391; ‘Overall, our findings highlight the need for further high-quality research, particularly appropriately powered randomized controlled trials, to generate robust evidence about the efficacy and safety of galactagogues to support evidence-based strategies to improve breastfeeding outcomes.’*

**7. Lines 268-270: Would the authors have data on which ethnic group used which galactagogue? (eg was ginger more popular with the Chinese?) It would be interesting it this data was available.**

*Response 7: Unfortunately our survey did not include questions about ethnicity, so we cannot address this question.*

**Reviewer #2: Thank you for the opportunity to review this interesting and well-written manuscript. It reports important findings resulting from a technically sound piece of scientific research. I have provided some minor suggestions and comments for consideration.

Introduction
1. Line 48: Please consider minor revision of the important first sentence with respect to the word "benefit".
Language used to describe lactation can have unintended interpretations. As noted in the third sentence, lactation is a phase of the reproductive cycle that statistics show is not functioning to recommendations. The description of breastfeeding as conferring “benefits” is problematic because it can be interpreted to imply that lactation is a beneficial optional extra, rather than a phase of the reproductive cycle that is the biologic norm (for example, we don’t typically talk about the “benefits” of effective heart function). Health outcomes are poorer for both mother and infant if this biologic norm is not sustained to recommendations.
Line 49: For similar reasons, please also consider changing the word “promote” to “recommend”.**
*Response 1: Line 51 has been amended to reflect your comments; ‘Breastfeeding is widely recognised to promote lifelong health for both the mother and infant’*

*Line 53 has been amended; ‘International recommendations are exclusive breastfeeding…’*

**2. Line 60: The reference cited does not cite evidence for association of correct positioning and latch with improvement in milk synthesis. Suggest alternative of ensuring maximal breast drainage (such as via increased breastfeed frequency +/- hand expression and/or pumping) as an evidence-based example of a strategy that increases milk synthesis.

Dewey KG, Lönnerdal B. Infant self‐regulation of breast milk intake. Acta Paediatrica. 1986;75(6):893-8.

Daly SE, Hartmann PE. Infant demand and milk supply. Part 2: The short-term control of milk synthesis in lactating women. Journal of Human Lactation. 1995;11(1):27-37.

Morton J, Hall JY, Wong RJ, Thairu L, Benitz WE, Rhine WD. Combining hand techniques with electric pumping increases milk production in mothers of preterm infants. Journal of perinatology : official journal of the California Perinatal Association. 2009;29(11):757-64.**

*Response 2: The Morton et al 2009 reference has been added to line 64 references.*

**3. Line 62: Breast milk supply includes consideration of both maternal milk synthesis and transfer of milk to the infant via breastfeeding or breast milk feeding. Suggest replacement of “supply” with “synthesis” as galactogogues only have the possibility of affecting maternal synthesis.**
*Response 3: The reviewer raises an interesting point. We acknowledge there is no universally accepted definition of galactagogues. In light of the comment we have changed the term ‘supply’ to ‘production’. Changes have been made to line 22; ‘substances thought to increase breast milk production’ and 66; ‘galactagogues – the term used to describe substances thought to promote or increase breast milk production’.* **Methods
4. Inclusion of the survey as an appendix or supplement would be very helpful.**

*Response 4: As above, the survey will be included as a supporting file.*

*The following has been added at line 134; ‘The complete survey is available as a supporting file (SI File 1).’ And a copy of the survey will be uploaded as a supporting file.*

**Results
5. Table 2: One bracket missing (Fennel; child’s age at survey 12+months)**

*Response 5: This has been amended in the table.*

**6. Line 199: Some text missing**

*Response 6: Missing text has been addressed, as per the Editor’s comments;*

*The following amendment has been made to line 218-220, where the underlined text has been added; ‘The proportion of women reporting commencing individual galactagogues within the first seven days postpartum, ranged from 4 to 67%.’*

**7. Figures 2 & 5: For me, the inclusion of colour assists with interpretation of these figures, however, perhaps consider ensuring that colour selection is colourblind-friendly.**

*Response 7: Figure 2 and 5 have been updated to a colourblind friendly scheme.*

**Discussion
8. Line 240: Objectively, this study is surveying the experience relating to galactogogue use during lactation function. Suggest deletion of “experience” descriptor for lactation.**

*Response 8: This has been amended to remove the term ‘experience’.*

**9. General comments for consideration, but not essential for inclusion:**
**These findings suggest none of the galactogogues are being used in a way that meets minimum quality use of medicine principles for safety and efficacy.**

**First, there is the lack of appropriate diagnosis to determine whether maternal concerns regarding lactation insufficiency are actual or just perceived and, if actual, to investigate the cause.**

**If lactation insufficiency is actual, this can be caused by a number of factors, including infant factors resulting in ineffective milk removal that subsequently cause down-regulation of milk synthesis (as stated in the introduction). This is of course further complicated by the lack of objective tests to assess milk production available in routine clinical practice.**

**Second, there is generally poor or absent evidence for understanding of galactogogue mechanism of action. Even domperidone, where the mechanism is known, is presumably commenced without investigation to determine whether low plasma prolactin is the cause of lactation insufficiency. Further, interpretation of plasma prolactin measurement itself is complicated by the absence of reference ranges for plasma prolactin in lactating women.**

**How concerning that maternal galactogogue use is so prevalent given that the cause (if lactation insufficiency is actually present) may not even be due to any disruption of maternal physiology, thus rendering the galactogogue without rationale for use and giving greater weight to any incidence of adverse effects. Clearly, women are worried about the adequacy of their breastmilk production and better strategies need to be developed to deliver effective support.**

**Well done, a thought-provoking study.**

*Response 9:*

*An amendment has been made to line 276 ‘discontinuing breastfeeding due to concerns about their milk supply. It is uncertain whether concerns related to breast milk supply were real or perceived, determining which has been often recognised as a common challenge within clinical practice settings (6).’*

*An amendment has been made in the concluding remarks to reflect some of the general comments above; ‘Overall, our findings highlight the need for further high-quality research, particularly appropriately powered randomized controlled trials, to generate robust evidence about the efficacy and safety of galactagogues to support evidence-based strategies to improve breastfeeding outcomes.’*

*Some of the comments above are also reflected in lines 333-339; ‘The fact that 1 in 6 respondents started using various galactagogues within the first seven days postpartum raises potential concerns, particularly given the challenge of assessing the adequacy of breast milk production in the early postpartum period (34, 35). These findings may indicate that women may be turning to galactagogues prophylactically (without actually having low breast milk supply) or using them as early treatments before trying non-pharmacological strategies. While 71% of women reported seeing a lactation consultant, we do not know when this occurred relative to the commencement of galactagogues.’*

Thank you again for considering our revised manuscript.

Yours sincerely

On behalf of the authors

**Dr Luke Grzeskowiak**

PhD, BPharm(Hons), GCertClinEpid, FSHP

*SAHMRI Women and Kids*

*Level 7 Clarence Rieger Building*

*Women’s and Children’s Hospital*

*72 King William Road, North Adelaide, SA, 5006*

[*Luke.Grzeskowiak@flinders.edu.au*](mailto:Luke.Grzeskowiak@flinders.edu.au)
